# Retraction: Impact of miR-223-3p and miR-2909 on inflammatory factors IL-6, IL-1ß, and TNF-α, and the TLR4/TLR2/NF-κB/STAT3 signaling pathway induced by lipopolysaccharide in human adipose stem cells

**DOI:** 10.1371/journal.pone.0341288

**Published:** 2026-01-21

**Authors:** The PLOS One Editors

Following the publication of this article [1] concerns were raised about results presented in Figs 1, 6-8, 10, and 11. Specifically, the following panels appear to overlap:

- Fig 1J β-actin lanes 1–6 and Fig 8J β-actin
- Fig 6D STAT3, Fig 7K STAT 3 lanes 3–4, and Fig 8A STAT3
- Fig 6D β-actin, Fig 7K β-actin lanes 3–4, and Fig 8A β-actin
- Fig 6L KLF4 and Fig 11J KLF4 lanes 3–4
- Fig 6L β-actin and Fig 11J β-actin lanes 4–5
- Fig 8J TLR4 lanes 2–6 and Fig 10H STAT3
- Fig 8J TLR4 lanes 5–6, Fig 10H STA3 lanes 4–5, and Fig 10G STAT 3
- Fig 8J TLR2 lanes 2–6 and Fig 10H pSTAT3
- Fig 10C STAT3 and Fig 10D pSTAT3 lanes 2–3
- Fig 10C β-actin and Fig 10D β-actin lanes 2–3
- Fig 10G β-actin and Fig 10H β-actin lanes 4–5

The authors did not respond to the journal’s questions pertaining to these concerns.

In light of the unresolved concerns, which call into question the reliability and integrity of the published results, the *PLOS One* Editors retract this article.

All authors either did not respond directly or could not be reached.
